# Patterns of Homotypic and Heterotypic Continuity Between ADHD Symptoms, Externalising and Internalising Problems from Age 7 to 15

**DOI:** 10.1007/s10802-019-00592-9

**Published:** 2019-11-08

**Authors:** Ingrid Obsuth, Aja Louise Murray, Simona Di Folco, Denis Ribeaud, Manuel Eisner

**Affiliations:** 1 Department of Clinical and Health Psychology, University of Edinburgh, Old Medical School, Teviot Place, Edinburgh, EH8 9AG Scotland; 2 Institute of Criminology, University of Cambridge, Sidgwick Ave, Cambridge, Cambridge, CB3 9DA UK; 3 Department of Psychology, University of Edinburgh, 7 George Square, Edinburgh, EH8 9JZ Scotland; 4 Jacobs Center for Productive Youth Development, University of Zürich, Andreasstrasse 15, 8050 Zürich, Switzerland

**Keywords:** Homotypic continuity, Heterotypic continuity, ADHD, Internalising, Externalising, Longitudinal

## Abstract

ADHD presents a serious community-health problem through its links to a wide range of negative outcomes. These outcomes are exacerbated when ADHD symptoms co-occur with other mental health problems. Research evidence suggests high rates of co-comorbidity with a range of problems. However, there is a paucity of longitudinal research that examines the predictive links between ADHD symptoms and symptoms of other mental health problems. We examined a cross-lagged autoregressive model in order to assess homotypic and heterotypic continuity between ADHD symptoms, aggressive behavior, non-aggressive behavior problems and anxiety/depression in a community-based sample of 1571 youth (761 female, 810 male) assessed annually from age 7 to 13 and again at age 15. Consistently significant correlations between each pair of problem behaviors provided support for concurrent comorbidity. Furthermore, significant autoregressive pathways provided support for homotypic continuity. Support for heterotypic continuity was limited to ADHD symptoms predicting both aggressive behavior and non-aggressive behavior problems, but not vice versa. Our study highlights the importance of focusing on ADHD symptoms to identify children at risk not only for continued ADHD symptomatology but also a range of externalizing behavior problems including different types of aggression and non-aggressive behavior problems, such as rule-breaking. Identifying these patterns in a community-based sample provides support for the possibility of early identification of risk for a range of problem  behaviors.

Attention deficit hyperactivity disorder (ADHD) is one of the most common disorders in school-aged children and it is characterised by hyperactivity, impulsiveness, and/or inattention (APA 2013). It presents a community-health problem when untreated as it has been linked to a wide range of negative long-term outcomes such as low educational attainment, unplanned pregnancy, unemployment, substance abuse and criminality (e.g., Owens et al. 2017). Moreover, ADHD and ADHD-related symptoms frequently co-occur with other psychiatric disorders, with the incidence of comorbidity ranging from 50% to 90% (Reale et al. 2017; Kadesjö and Gillberg 2001). Available studies suggest that when the symptoms are persistent and comorbid with symptoms of other mental health problems the outcomes are more varied and more serious (e.g., Biederman 2004). The presence of an additional disorder also markedly influences the prognosis, treatment, and outcomes of therapeutic interventions (Sibley et al. 2011).

However, the majority of the studies investigating ADHD comorbidity have been carried out based on concurrently assessed clinical samples. For instance, Reale et al. (2017) utilized a clinical sample of youths registered in 18 ADHD treatment centres across Italy and found that of the 2861 children and adolescents, 67% (*n* = 1919) met criteria for ADHD; of those 34% received a diagnosis of ADHD alone, while the other 66% met criteria for at least one other comorbid diagnosis. Similar findings were reported by Jensen and Steinhausen in 2015 in a sample of 14,825 Danish 4- to 17-year-old patients. While fewer studies have examined comorbidity utilising non-clinical samples, the available studies also suggest similar findings. For instance, Kadesjö and Gillberg (2001) reported that 87% percent of their sample of Swedish 7-year olds drawn from the general population who met criteria for ADHD also met criteria for one more disorder, and 67% met criteria for two or more additional diagnoses. The highest comorbidity (60%) was reported with oppositional defiant disorder (ODD).

Past longitudinal research that has examined the links between ADHD and other disorders has explored ADHD symptoms as a risk for the development of other problem behaviors but rarely vice versa (e.g., Yoshimasu et al. 2012). For instance, longitudinal studies have found that children who met diagnostic criteria for ADHD in childhood (ages 5–11) were in adolescence more likely to exhibit oppositional defiant behaviors, anxiety/depression as well as involvement with the juvenile justice system (e.g., Bussing et al. 2010). Studies on children and/or adolescents with ADHD followed up in adulthood showed that antisocial personality disorder, criminality, and drug misuse are among their externalizing outcomes (e.g., Klein et al. 2012). These finding may be interpreted in light of the underlying neurodevelopmental deficits of ADHD related to difficulties in emotion regulation and functional impairments that may place young people at risk for later problem behaviors particularly in the context of other risk factors (e.g., parenting practices, peer problems; e.g., Anastapoulos et al. 2010).

Complementary to this interpretation Beauchaine and McNulty (2013) described longitudinal comorbidities as, what they refer to, ontogenetic processes. According to their theory, early-onset externalizing problems may lead to other mental health problems via complex longitudinal transactions between individual vulnerabilities (e.g., genetic factors) and environmental risk factors (e.g., deviant peers). However, little is known about whether earlier problem behaviors may influence later ADHD or ADHD-like features. This is rather surprising given that externalizing behavior problems, including aggressive behavior and ODD-like behaviors (such as defiance), as well as ADHD or ADHD-like symptoms are often observed and first diagnosed very early in life (as early as 3-years of age; e.g., Kadesjö et al. 2001). Moreover, increasing recognition that ADHD symptoms may not reach clinically significant levels until adolescence (e.g., Caye et al. 2016) has highlighted the need to examine potential precipitating factors in the form of personality characteristics, contextual factors as well as other problem behaviors, such as externalizing problems, earlier in development (e.g., Castellanos 2015).

Yet, based on our review of the literature, only three studies examined the bi-directional links between ADHD and externalizing problems over time. Most recently, Kuja-Halkola et al. (2015) examined a birth cohort of 2600 Swedish twins assessed at ages 8–9, 13–14, 16–17, and 19–20 and found that comorbidity increased over time, and externalizing traits in middle childhood (age 8–9) predicted ADHD-like traits in early adolescence (age 13–14), whereas the reverse was not supported. On the other hand, ADHD-like traits in late adolescence (age 16–17) predicted externalizing traits in early adulthood (age 19–20) but not vice versa. Another study on clinic-referred boys suggested that childhood behavior problems predicted later ADHD symptoms, when early levels of ADHD were taken into account, but not vice versa (Lahey et al. 2002). Conversely, another study on boys screened positive for conduct problems at age 6 and 7 did not show any significant prediction in terms of hyperactivity ratings at age 16–18 (Taylor et al. 1996). The limited findings of these studies present an inconsistent and incomplete picture of the mutual influence of ADHD and other externalizing problems. However, they point to potential developmental changes in the links and continuity of these symptoms.

Much less is known about the reciprocal developmentally predictive links between ADHD and other, non-externalizing, disorders. One exception is a recent study carried out by Shevlin et al. (2017), that examined homotypic (within-disorder) and heterotypic (between-disorders) continuity of eight psychiatric disorders including ADHD, measured at age 7.5 and again at age 14 years, in a UK child cohort comprising the Avon Longitudinal Study of Parents and Children (ALSPAC, *N* = 4815). The authors found strong support for homotypic continuity, that is, each disorder predicted itself seven years later. They also found strong support for heterotypic continuity, particularly within the higher order domains of externalizing and internalizing problems; that is, one disorder predicted another disorder seven years later within the same domain. They also found support for some cross-domain heterotypic continuity, the strongest one being a significant link between ADHD symptoms at age 7.5 and internalizing disorders at age 14. According to the authors, these findings are consistent with the ‘dual failure model’ (e.g., Capaldi and Shortt 2003) of developmental psychopathology, in which persistent attention problems and hyperactivity may elicit negative responses from others, including peers, parents, teachers. In turn, these responses may cause distress in the child that is eventually expressed as an internalizing problem. This interpretation of comorbidity is consistent with developmental theories, such as the ‘cascade model’ of development (Masten and Cicchetti 2010) that suggests that links between symptoms of different problem behaviors can emerge over time without necessarily having a common cause. In this way, one disorder may have a causal effect on another one. In line with these perspectives, it may be important to consider comorbidity in a sequential way, in addition to examining it concurrently.

In summary, studies investigating ADHD consistently show that its concurrent comorbidity rate is particularly high with externalizing behavior problems, such as conduct problems and disruptive behavior problems (Jensen and Steinhausen 2015; Reale et al. 2017). However, relatively little is known about the longitudinal links or the degree to which ADHD symptoms predict other disorders from childhood into late adolescence and vice versa. A better understanding of these dynamic links and identification of the conditions that may predict others from an early age will not only inform the early identification, prevention, and intervention practices related to ADHD, but also further illuminate the structure of psychopathology (Forbes et al. 2016).

Therefore, the aim of the current study was to build on previous research and examine the patterns of homotypic and heterotypic continuity of ADHD symptoms and other externalizing problems, as well as internalizing problems assessed annually from age 7 to age 13 and again at age 15. Wittchen et al. (2009) suggest that different underlying structures of co-morbidity may manifest at different developmental stages. Given the remarkable developmental changes occurring during this time-period in terms of biological, cognitive, socio-emotional development, it is important to examine the patterns of homotypic and heterotypic continuity of psychopathology more closely. Annual assessments allow for the identification of changing patterns that may point to potential entry points for targeted prevention and intervention at specific ages from entry to school through adolescence.

We examined the reciprocal longitudinal links between ADHD symptoms and two aspects of externalizing behavior problems – namely, aggressive behavior and non-aggressive behavior problems/rule-breaking. Past research has identified these two behaviors as distinct aspects of externalizing problems (e.g., Forbes et al. 2016) with specific aetiologies. While aggressive behavior has been described as a highly heritable and stable characteristic with onset often in early childhood, rule-breaking behavior has been identified as having a stronger environmental influence, less stability and highest onset and frequency during adolescence (e.g., Burt 2012). Due to these differences, it was important to examine them separately. Furthermore, instead of focusing on diagnoses or diagnostic cut-offs of ADHD or any of the other disorders, we were interested in examining the continua of different sets of difficulties. To this end, we utilized dimensional scores of the assessed socio-behavioral problems. This approach has been supported by previous research, which suggests that most variation in psychopathology is dimensional (Haslam et al. 2012). Moreover, dimensional scores are more sensitive to capturing even milder manifestations of psychopathology (Finsaas et al. 2018).

Based on the above-reviewed literature related to the longitudinal links between these behavior problems, we predicted homotypic continuity of each of the disorders. As ADHD is often categorised as an externalizing problem, we expected the links between ADHD symptoms and each of the externalizing problems to yield greater effect sizes than ADHD symptoms and internalizing problems. In addition, we explored heterotypic continuity, namely, longitudinal links between ADHD symptoms and aggression, non-aggressive behavior, as well as anxiety/depression. While the links from externalizing problems to ADHD are largely unexplored, consistent with the available literature and theory described above, we anticipated greater effect sizes for links from ADHD to externalizing problems than vice-versa.

To date, very few studies have examined comorbidity, or heterotypic continuity patterns longitudinally in child and adolescent general population samples. Furthermore, to our knowledge, no studies have examined these patterns year-to-year over a period of eight years to assess ‘short-term’ heterotypic continuity. Population-based studies are key to provide general patterns free of referral bias, local clinical practice and availability. In order to reduce the likelihood of the adverse social and emotional outcomes of childhood problems, it is crucial to screen for comorbid conditions and identify which conditions may predict others from an early age. It is equally important to understand at what age links between conditions or symptoms emerge and whether these links change over time. This also has the potential to reduce the burden and costs on society by providing interventions aimed at targeting the main, or ‘driving’, condition.

## Method

### Participants

The data were drawn from an ongoing combined cohort and intervention study, the Zurich Project on Social Development from Childhood to Adulthood (z-proso). Ethics approval for the study was provided by the Ethics Committee at the Faculty of Arts and Social Sciences of the University of Zurich. The target sample at the initial assessment was 1675 first graders from 56 public elementary schools. Schools were selected according to a stratified random sampling procedure that took into account school location and size. All children who entered first grade (aged ~7) in 2004 in one of these schools were invited to participate via their parents. Parents provided informed consent on behalf of their child until age 11, after which point the participants themselves provided consent. The current study includes 1571 (761 girls, 810 boys) participants with teacher-reported symptom data available for at least one measurement point. This represents 94% of the baseline target sample. Data was collected at eight time points spanning the duration of compulsory schooling in Zurich, Switzerland (child age 7–16). These are labelled according to the children’s age at each point rounded to the nearest whole number. The mean age and number of participants at each data collection point were 7.45 (*n* = 1349), 8.23 (*n* = 1343), 9.21 (*n* = 1294), 10.70 (*n* = 1269), 11.60 (*n* = 1064), 12.63 (*n* = 977), 13.88 (*n* = 1266), and 15.68 (*n* = 1288). Approximately 11% of the children were born outside of Switzerland. At baseline, 78% lived with both of their biological or adoptive parents, 20% with their biological mother only, and 2% with their biological father only, with foster parents or in residential care. Notably, 46% of the children had both parents who were born outside of Switzerland representing more than 80 countries of origin and contributing to an ethnically diverse sample. Twenty-five percent of the primary caregivers had little or no secondary education, 30% had vocational training, 29% had attended full-time vocational school, had a baccalaureate degree or advanced vocational diploma, and 16% had a university degree. In terms of household finances, 18% reported experiencing financial difficulties in the last year. Further information on the sample can be found at: http://www.jacobscenter.uzh.ch/en/research/zproso/aboutus.html as well as previous publications (e.g., Eisner and Ribeaud 2007).

### Measures

The current study relied on teacher reported data. For the majority of children, the same teacher taught and provided ratings for them from Grade 1 to 3 (at ages 7, 8 and 9) of primary school. Another teacher provided ratings from Grade 4 to 6 (at ages 10, 11, and 12). Following this, children transitioned into secondary school, where a third teacher provided the ratings at ages 13 and 15. The number of teachers who provided ratings each year were 113, 148, 217, 274, 265, 258, 366, and 423, respectively. They provided ratings for 1 to 24 children, with an average ranging from 6 to 12 over all the data collection points. At each time point, the research team approached the so called ‘class teacher’ to complete the questionnaire. Class teachers are the Swiss equivalent of ‘home room teacher’ in US and Canada, with responsibilities to oversee each student’s progress. They are also the contact person for personal issues and liaise with the students’ parents. These teachers are also responsible for all of the teaching in primary school (up to age 13) and at least some of the teaching after that. The study requirement was that they have at least 4 lessons weekly with each rated student. If the ‘class teacher’ taught the class for less than 4 lessons per week, the rule was that the German language teacher should complete the questionnaires.

To rate children’s behavior, teachers completed the Social Behavior Questionnaire (SBQ; Tremblay et al. 1991). The SBQ is composed of around 45 items, depending on the measurement wave because new items were added and others removed to maintain developmental appropriateness. It is a paper and pencil questionnaire rated on a 5-point Likert scale from never = ‘0’ to very often = ‘4’. The SBQ is used to rate children’s psychosocial functioning across five sub-scales mapping onto psychopathological profiles: ADHD, anxiety/depression, aggression, non-aggressive externalizing problems and prosocial behavior. Previous psychometric analyses have supported the criterion validity, factorial validity as well as developmental invariance of the SBQ and showed that it can reliably measure psychopathology from moderately low to very high trait levels (Murray et al. 2018).

For the purposes of the current study, we utilized the four subscales tapping symptoms of commonly occurring social behavioral problems. The Cronbach’s alphas for each of the subscales demonstrated excellent internal consistency over time. The *ADHD symptoms scale* included 8 items, such as “The child can’t sit still, is restless, hyperactive”; “The child is distractible, has trouble sticking to any activity”. (αs = 0.94, 0.95, 0.94, 0.95, 0.95, 0.95, 0.95, & 0.94; at each time point, respectively). The *non-aggressive externalizing problems scale* (NAEX) included 6 items, such as “The child destroys things belonging to his/her family, or other children”. (αs = 0.85, 0.84, 0.84, 0.86, 0.84, 0.83, 0.85, & 0.83). The *aggression scale* included 11 items tapping three forms of common types of aggression – physical, proactive and reactive. Examples of items include: “The child physically attacks people”, “The child threatens people”, “The child reacts in an aggressive manner when teased”, respectively (αs = 0.94, 0.93, 0.93, 0.93, 0.94, 0.93, 0.93, & 0.93). The *anxiety/depression scale* consisted of 7 items, such as “The child is worried”, “The child has trouble enjoying him/herself” (αs = 0.91, 0.91, 0.92, 0.91, 0.90, 0.91, 0.92, & 0.90). Anxiety and depression were combined into one subscale as they were conceptualised as manifestations of the same underlying disorder (e.g., Savage et al. 2015). The two sets of items are combined in other well-established measures of general psychopathology (e.g., CBLC; Achenbach 1991) thus combining them ensures comparability with studies utilising these measures. The combined anxiety/depression subscale has been validated in this format on this sample (Murray et al. 2018) and exploratory factor analyses and reliability coefficients supported a single factor. Table 1 presents descriptive statistics for the study variables at each assessment point.

**Table 1:** Descriptive statistics for study variables

Age (n)	ADHD	Aggression	NAEX	Anxiety/Depression
	Mean/SD	Mean/SD	Mean/SD	Mean/SD
Age 7 (*n* = 1349)	1.25/0.99	0.59/0.68	0.33/0.49	0.87/0.76
Age 8 (*n* = 1343)	1.10/0.98	0.55/0.64	0.31/0.48	0.79/0.73
Age 9 (*n* = 1294)	1.07/0.95	0.57/0.64	0.33/0.52	0.84/0.74
Age 10 (*n* = 1269)	1.11/0.99	0.54/0.69	0.27/0.47	0.90/0.74
Age 11 (*n* = 1064)	1.07/0.99	0.48/0.63	0.27/0.47	0.90/0.76
Age 12 (*n* = 977)	1.00/0.94	0.48/0.64	0.31/0.52	0.89/0.77
Age 13 (*n* = 1266)	1.05/0.94	0.36/0.54	0.23/0.44	0.87/0.76
Age 15 (*n* = 1288)	1.04/0.92	0.35/0.51	0.28/0.48	0.87/0.75

*SD* standard deviation, *NAEX* non-aggressive externalizing

### Data Analytic Approach

Subscales were calculated by averaging the individual item scores for each of the behaviors. To examine concurrent comorbidity, homotypic and heterotypic continuity over time, data analyses were conducted via cross-lagged regression models in a structural equation modelling (SEM) framework with the statistical software *Mplus 7.31* (Muthen and Muthen 2014), using robust maximum likelihood estimation (MLR) as it provides unbiased parameter estimates under the assumption of missing at random (MAR). Clustering by classes was dealt with by including it via the “cluster” command in the Mplus script estimating the model. All individuals with at least one data point for any given variable were included in the analyses.

First-order autoregressive and cross-lagged pathways of association along with concurrent correlations were simultaneously evaluated. In a first-order autoregressive model, variables are represented as predictors of themselves over time. Therefore, autoregressive pathways estimate the association between each of the behaviors at time t_n_ with the same behavior at the following assessment point, at time t_n + 1_. In other words, they adjust for past levels of the outcome in order to predict change in levels of the outcome over time. Consistent with previous research the autoregressive pathways were allowed to vary across time to allow for the changes in the level of influence that behaviors at time t_n_ have on the same behaviors at time t_n + 1_ as children grow older.

Cross-lagged models have been widely used in developmental research to assess bi-directional time-lagged relations (e.g., Defoe et al. 2013; Murray et al. 2017; Obsuth et al. 2015). The cross-lagged associations represent relations between one behavior at time t_n_ and another behavior at time t_n + 1_ as well as the reciprocal association between the two behaviors at time t_n_ and at time t_n + 1_. These effects were allowed to vary across time to examine change in the reciprocal association between behaviors from age 7 through to age 15. Concurrent residual correlations between pairs of behaviors at the same time of assessment were estimated and allowed to vary over time as were the residuals within construct variances.

In the first step of the analyses, we examined whether invariance across boys and girls can be assumed. Model invariance was assumed to be more parsimonious and was tested by comparing the fit indices of nested models: A model where all the regression weights were free to vary across boys and girls (allowing for sex differences), and a model in which these regression weights were constrained to be equal. Because the *χ*^*2*^ becomes increasingly sensitive with growing sample size (Marsh et al. 1988), it is presented for information but was not considered for evaluation of model fit. Instead, we used practical fit indexes to test for sex invariance and to assess the fit of each model. Following recommendation by Little (1997), model invariance can be assumed (a) if the overall model fit is acceptable, as indicated by relative fit indexes (e.g., if the CFI is approximately 0.90 or greater; and if the RMSEA is less than 0.05; McDonald and Ho 2002); (b) if the difference in model fit is negligible (e.g., ≤ 0.05 for the fit indices) after introduction of the equality constraints; and (c) if the justification for the accepted model is substantively more meaningful and the interpretation is more parsimonious than the alternative model. In addition, we followed recommendations by Rigdon (1996) and used the 95% confidence interval (CI) around the RMSEA to evaluate model fit and for nested model comparisons. Specifically, if the upper bound of the CI is equal to or lower than 0.05, a close fit of the model to the data can be assumed. Moreover, if the CIs of subsequent nested models overlap with those of preceding, less constrained models, the more parsimonious model is deemed acceptable.

Following the assessment of model fit and provided a good fit was identified, correlations between each pair of problem behaviors were examined to assess concurrent co-occurrence, or comorbidity, of problem behaviors at each time point. Next, the autoregressive pathways were interpreted to evaluate homotypic continuity of each problem behavior (ADHD symptoms, aggression, non-aggressive behavior problems and anxiety/depression) over time. Finally, to address the main goal of the study, the cross-lagged pathways were interpreted to evaluate heterotypic continuity, or prediction of different problem behaviors predicting each other over time.

## Results

### Overall Model Fit

**Fig. 1:**
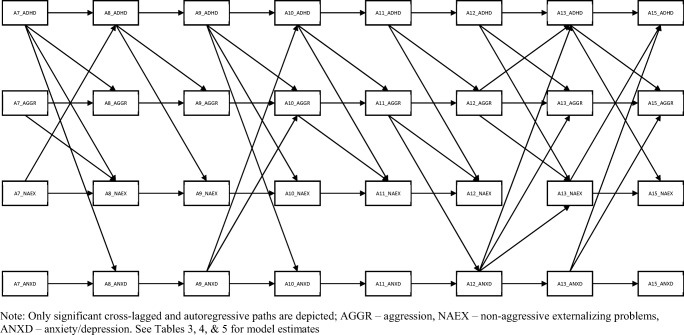
Autoregressive cross-lagged model from age 7 to 15 – only significant auto-regressive and cross-lagged paths

In the first step of the analyses, comparison of fit indices supported sex invariance (no significant sex differences) in the examined cross-lagged autoregressive model, which included eight time points and four mental health categories (ADHD symptoms, Anxiety/Depression, Aggression and Non-aggressive externalizing problems, see Table 2; Fig. 1). Given support for sex invariance, individual paths are interpreted for the combined sample (RMSEA = 0.04, 90% CI (0.03; 0.04), CFI = 0.96, NFI = 0.94).

**Table 2:** Summary of nested model tests regarding sex invariance and test based on the total/combined sample

	Unconstrained	Constrained to be equal	Combined sample
RMSEA	0.04	0.04	0.04
90% CI	0.04–0.04	0.04–0.04	0.03–0.04
CFI	0.96	0.94	0.96
NFI	0.94	0.93	0.94

The models were also run controlling for SES and yielded the same pattern of findings

*NFI* normed fit index, *CFI* comparative fit index, *RMSEA* root mean square of approximation

#### Concurrent Correlations – Comorbidity

The residual correlations between problem behaviors assessed concurrently were consistently statistically significant (see Table 3). They were consistent with small to medium effect sizes and pointed to comorbidity between each pair of problem behaviors but of varied strength over time and for different problems. The strongest association was found for Aggression and Non-aggressive externalizing problems, followed by the link between ADHD symptoms and Non-aggressive externalizing problems, comparable with the link between ADHD and Aggression. Smaller effects were found for the links between Anxiety/ Depression and each ADHD, Aggression and Non-aggressive externalizing problems.

**Table 3:** Model-based correlations between variables at each age

	Age 7	Age 8	Age 9	Age 10	Age 11	Age 12	Age 13	Age 15
ADHD x Aggr	0.57	0.43	0.49	0.51	0.51	0.36	0.47	0.50
ADHD x NAEX	0.63	0.47	0.49	0.53	0.53	0.45	0.49	0.56
ADHD x AnxD	0.36	0.27	0.32	0.35	0.37	0.23	0.34	0.29
Aggr x NAEX	0.70	0.57	0.57	0.63	0.59	0.49	0.61	0.69
Aggr x AnxD	0.34	0.24	0.32	0.26	0.27	0.20	0.31	0.34
AnxD x NAEX	0.38	0.25	0.28	0.30	0.28	0.20	0.27	0.30

All correlations were statistically significant at *p* < 0.001

*Aggr* aggression, *NAEX* non-aggressive externalizing problems, *AnxD* anxiety/depression

#### Autoregressive Relations – Homotypic Continuity

As expected, with only one exception, mental health problems at time t were significantly related to the same mental health problem at t_n + 1_ (see Table 4). The one exception was the non-significant link between Non-aggressive externalizing problems at age 13 and 15, all remaining links (age 7 to 8; 8 to 9; 9 to 10; 10 to 11; 11 to 12; 12 to 13) were significant (all *p*s < 0.001). The links at all ages were significant with respect to ADHD symptoms, Anxiety/Depression, and Aggression (all *p*s < 0.001), suggesting that previous behavior significantly, and consistently predicted the same future behavior.

**Table 4:** Auto-regressive pathways of each social-behavioral difficulty from t_n_ to t_n + 1_

Scale at t_n_ (age)	Scale at _tn + 1_ (age)	Estimate	S0.E0.	*p* value
ADHD (7)	ADHD (8)	0.68	0.04	<0.001
ADHD (8)	ADHD (9)	0.67	0.03	<0.001
ADHD (9)	ADHD (10)	0.54	0.03	<0.001
ADHD (10)	ADHD (11)	0.68	0.04	<0.001
ADHD (11)	ADHD (12)	0.76	0.03	<0.001
ADHD (12)	ADHD (13)	0.50	0.04	<0.001
ADHD (13)	ADHD (15)	0.62	0.03	<0.001
Aggression (7)	Aggression (8)	0.58	0.04	<0.001
Aggression (8)	Aggression (9)	0.54	0.04	<0.001
Aggression (9)	Aggression (10)	0.30	0.05	<0.001
Aggression (10)	Aggression (11)	0.50	0.07	<0.001
Aggression (11)	Aggression (12)	0.63	0.04	<0.001
Aggression (12)	Aggression (13)	0.42	0.06	<0.001
Aggression (13)	Aggression (15)	0.34	0.05	<0.001
NAEX problems (7)	NAEX problems (8)	0.51	0.05	<0.001
NAEX problems (8)	NAEX problems (9)	0.49	0.07	<0.001
NAEX problems (9)	NAEX problems (10)	0.27	0.06	<0.001
NAEX problems (10)	NAEX problems (11)	0.39	0.05	<0.001
NAEX problems (11)	NAEX problems (12)	0.43	0.06	<0.001
NAEX problems (12)	NAEX problems (13)	0.06	0.08	0.403
NAEX problems (13)	NAEX problems (15)	0.24	0.06	<0.001
Anxiety/Depression (7)	Anxiety/Depression (8)	0.57	0.03	<0.001
Anxiety/Depression (8)	Anxiety/Depression (9)	0.57	0.03	<0.001
Anxiety/Depression (9)	Anxiety/Depression (10)	0.23	0.04	<0.001
Anxiety/Depression (10)	Anxiety/Depression (11)	0.48	0.03	<0.001
Anxiety/Depression (11)	Anxiety/Depression (12)	0.62	0.03	<0.001
Anxiety/Depression (12)	Anxiety/Depression (13)	0.29	0.04	<0.001
Anxiety/Depression (13)	Anxiety/Depression (14)	0.42	0.03	<0.001

Estimate – standardized auto-regressive paths; S.E. – standard error; NAEX – non-aggressive externalizing problems. The models were also run controlling for SES and yielded the same pattern of findings

#### Cross-Lagged Relations – Heterotypic Continuity

Next, we examined the cross-lagged effects between each pair of behaviors (see Table 5 for all estimates). Higher levels of teacher observed ADHD symptoms at time t_n_ were consistently and significantly linked to higher levels of teacher-observed Non-aggressive externalizing problems at time t_n + 1_ from age 7 through 15. However, higher levels of Non-aggressive externalizing problems at time t_n_ were significantly linked to higher levels of ADHD symptoms only at ages 7 to 8 and 13 to 15.

**Table 5:** Cross-lagged (longitudinal) pathways between the different social-behavioral difficulties from t_n_ to t_n + 1_

Scale 1 at t_n_ (age)	Scale 2 at _tn + 1_ (age)	Estimate	S.E.	*p* value
ADHD (7)	Aggression (8)	0.10	0.03	0.001*
ADHD (8)	Aggression (9)	0.10	0.03	0.004*
ADHD (9)	Aggression (10)	0.18	0.05	<0.001*
ADHD (10)	Aggression (11)	0.14	0.05	0.002*
ADHD (11)	Aggression (12)	0.12	0.04	0.001*
ADHD (12)	Aggression (13)	0.13	0.05	0.005*
ADHD (13)	Aggression (15)	0.21	0.03	<0.001*
Aggression (7)	ADHD (8)	0.01	0.03	0.701
Aggression (8)	ADHD (9)	0.05	0.04	0.129
Aggression (9)	ADHD (10)	0.04	0.04	0.254
Aggression (10)	ADHD (11)	0.08	0.05	0.095
Aggression (11)	ADHD (12)	0.02	0.04	0.608
Aggression (12)	ADHD (13)	0.13	0.05	0.005*
Aggression (13)	ADHD (15)	0.01	0.03	0.832
ADHD (7)	NAEX problems (8)	0.10	0.04	0.015
ADHD (8)	NAEX problems (9)	0.15	0.04	<0.001*
ADHD (9)	NAEX problems (10)	0.21	0.04	<0.001*
ADHD (10)	NAEX problems (11)	0.18	0.04	<0.001*
ADHD (11)	NAEX problems (12)	0.23	0.04	<0.001*
ADHD (12)	NAEX problems (13)	0.19	0.05	<0.001*
ADHD (13)	NAEX problems (15)	0.25	0.04	<0.001*
NAEX problems (7)	ADHD (8)	0.09	0.03	0.014
NAEX problems (8)	ADHD (9)	0.05	0.05	0.249
NAEX problems (9)	ADHD (10)	0.07	0.04	0.067
NAEX problems (10)	ADHD (11)	0.02	0.04	0.709
NAEX problems (11)	ADHD (12)	0.05	0.04	0.253
NAEX problems (12)	ADHD (13)	0.08	0.05	0.138
NAEX problems (13)	ADHD (15)	0.09	0.04	0.024
ADHD (7)	Anxiety/Depression (8)	0.11	0.04	0.003*
ADHD (8)	Anxiety/Depression (9)	0.05	0.04	0.203
ADHD (9)	Anxiety/Depression (10)	0.11	0.05	0.018
ADHD (10)	Anxiety/Depression (11)	−0.00	0.04	0.957
ADHD (11)	Anxiety/Depression (12)	0.03	0.04	0.466
ADHD (12)	Anxiety/Depression (13)	−0.04	0.05	0.415
ADHD (13)	Anxiety/Depression (15)	−0.01	0.04	0.789
Anxiety/Depression (7)	ADHD (8)	0.03	0.02	0.083
Anxiety/Depression (8)	ADHD (9)	0.03	0.03	0.337
Anxiety/Depression (9)	ADHD (10)	−0.08	0.03	0.005*
Anxiety/Depression (10)	ADHD (11)	−0.03	0.03	0.274
Anxiety/Depression (11)	ADHD (12)	−0.00	0.03	0.928
Anxiety/Depression (12)	ADHD (13)	−0.12	0.03	<0.001*
Anxiety/Depression (13)	ADHD (15)	−0.08	0.02	0.001*
NAEX problems (7)	Aggression (8)	0.05	0.05	0.360
NAEX problems (8)	Aggression (9)	0.08	0.06	0.156
NAEX problems (9)	Aggression (10)	0.07	0.05	0.203
NAEX problems (10)	Aggression (11)	0.03	0.06	0.679
NAEX problems (11)	Aggression (12)	0.06	0.05	0.236
NAEX problems (12)	Aggression (13)	0.06	0.06	0.364
NAEX problems (13)	Aggression (15)	0.04	0.05	0.372
Aggression (7)	NAEX problems (8)	0.07	0.03	0.031
Aggression (8)	NAEX problems (9)	0.06	0.05	0.202
Aggression (9)	NAEX problems (10)	0.03	0.05	0.577
Aggression (10)	NAEX problems (11)	0.11	0.05	0.014
Aggression (11)	NAEX problems (12)	0.15	0.05	0.001*
Aggression (12)	NAEX problems (13)	0.34	0.05	<0.001*
Aggression (13)	NAEX problems (15)	0.04	0.05	0.452
Anxiety/Depression (7)	Aggression (8)	0.05	0.03	0.112
Anxiety/Depression (8)	Aggression (9)	-0.03	0.03	0.385
Anxiety/Depression (9)	Aggression (10)	-0.08	0.03	0.014
Anxiety/Depression (10)	Aggression (11)	−0.00	0.03	0.975
Anxiety/Depression (11)	Aggression (12)	−0.01	0.03	0.633
Anxiety/Depression (12)	Aggression (13)	−0.08	0.03	0.004*
Anxiety/Depression (13)	Aggression (15)	−0.06	0.02	0.021
Aggression (7)	Anxiety/Depression (8)	0.04	0.04	0.269
Aggression (8)	Anxiety/Depression (9)	0.03	0.04	0.510
Aggression (9)	Anxiety/Depression (10)	−0.02	0.05	0.721
Aggression (10)	Anxiety/Depression (11)	0.04	0.05	0.412
Aggression (11)	Anxiety/Depression (12)	0.08	0.04	0.050
Aggression (12)	Anxiety/Depression (13)	0.06	0.05	0.234
Aggression (13)	Anxiety/Depression (15)	−0.02	0.04	0.533
Anxiety/Depression (7)	NAEX problems (8)	0.02	0.03	0.554
Anxiety/Depression (8)	NAEX problems (9)	0.02	0.03	0.554
Anxiety/Depression (9)	NAEX problems (10)	−0.03	0.03	0.361
Anxiety/Depression (10)	NAEX problems (11)	-0.02	0.03	0.604
Anxiety/Depression (11)	NAEX problems (12)	0.04	0.04	0.289
Anxiety/Depression (12)	NAEX problems (13)	−0.07	0.03	0.029
Anxiety/Depression (13)	NAEX problems (15)	-0.04	0.03	0.166
NAEX problems (7)	Anxiety/Depression (8)	−0.02	0.04	0.680
NAEX problems (8)	Anxiety/Depression (9)	0.06	0.05	0.197
NAEX problems (9)	Anxiety/Depression (10)	0.02	0.05	0.665
NAEX problems (10)	Anxiety/Depression (11)	0.07	0.04	0.093
NAEX problems (11)	Anxiety/Depression (12)	−0.01	0.06	0.834
NAEX problems (12)	Anxiety/Depression (13)	−0.02	0.05	0.752
NAEX problems (13)	Anxiety/Depression (15)	0.07	0.05	0.149

Estimate – standardized estimates of cross-lagged regression paths; S.E. – standard error; NAEX – non-aggressive externalizing problems

*Significant estimates following Bonferroni correction for family-wise error; *p* = 0.007 (0.05/7 – the number of regressions in each family of tests)

Higher levels of teacher observed ADHD symptoms at time t_n_ were also consistently and significantly linked to higher levels of teacher-observed Aggression at time t_n + 1_ from age 7 through 15. However, higher levels of Aggression at time t_n_ were significantly linked to higher levels of ADHD symptoms only at ages 12 to 13. Furthermore, higher levels of ADHD symptoms at time t_n_ were significantly linked to higher levels of Anxiety/Depression only at ages 7 to 8 and 9 to 10. Higher levels of Anxiety/Depression at time t_n_, on the other hand, were linked to lower levels of ADHD symptoms at ages 9 to 10, 12 to 13 and 13 to 15.

Higher levels of Anxiety/Depression at time t_n_ were also significantly linked to lower levels of Aggression at ages 9 to 10, 12 to 13 and 13 to 15 but Aggression at time t_n_ was only significantly related to higher levels of Anxiety/Depression at age 11 to 12. Similarly, higher levels of Anxiety/Depression at time t_n_ were also significantly linked to lower levels of Non-aggressive externalizing problems at age 12 to 13 but Non-aggressive externalizing problems at t_n_ were not significantly related to Anxiety/Depression.

Finally, Aggression at time t_n_ was also related to higher levels of Non-aggressive externalizing problems at ages 7 to 8, 10 to 11, 11 to 12 and 12 to 13. However, Non-aggressive externalizing problems at t_n_ were not significantly related to Aggression at any age.

## Discussion

This study investigated the concurrent links and longitudinal paths between ADHD symptoms, aggression, and non-aggressive externalizing behavior, as well as anxiety and depression from childhood to adolescence. It built on previous research pointing to high concurrent comorbidity, and homotypic as well as heterotypic continuity, particularly among disorders classified in the same higher order domain (externalizing vs. internalizing; e.g., Shevlin et al. 2017). Our study expanded on previous research by including eight assessment points from age 7 to 15 to better capture potential year-to-year changes over this period of development. Understanding the patterns of change in the mutual influence between these sets of problem behaviors over a period of time has the potential to inform timely identification of problems and allow better informed treatment planning.

### Concurrent Comorbidity

Consistent with previous studies, our findings provided further evidence for concurrent comorbidity or co-occurrence for each pair of measured problem behaviors at each point of data collection. These findings are in line with interpretations of comorbidity as an artefact of shared aetiology that may account for up to 50% in the overlap between mental health disorders (e.g., Caspi et al. 2013; ‘50% rule’). The links were strongest between the two types of assessed externalizing behavior problems, followed in strength by their links with ADHD symptoms, and weakest with anxiety/depression, so the findings provide some support for the externalizing and internalizing distinction.

### Homotypic Continuity

Similarly, homotypic continuity was evident consistently across time and problem behaviors; in 27 of the 28 assessed pathways. The one exception was a pathway from age 12 to 13 in non-aggressive externalizing problems. Predominant homotypic continuity is consistent with previous research, suggesting that past behavior is one of the best predictors of the same future behavior, particularly without intervention. This notion has been relatively well established in criminological research in relation to criminality (e.g., Farrington 1995), as well as psychological studies dealing with aggressive behavior (e.g., Eivers et al. 2012). Our findings provide further support for this supposition, particularly in the context of non-aggressive externalizing problems, ADHD symptoms as well as anxiety/ depression. These results highlight the importance of identifying children who experience a wide range of difficulties and may benefit from early intervention to prevent escalation.

### Heterotypic Continuity

More importantly, while controlling for homotypic continuity, we examined the cross-lagged (predictive) bidirectional links between each of the four problem behaviors measured at the eight time points from age 7 to 15. Of the 84 possible pathways of heterotypic continuity, only 31 were statistically significant. Interestingly, ADHD symptoms consistently predicted aggressive behavior as well as non-aggressive behavior problems in every consecutive assessment/wave from age 7 to 15. However, the reverse was not the case consistently, as ADHD symptoms were predicted by aggression only from age 12 to 13 and by non-aggressive behavior problems from age 7 to 8 and 13 to 15. Previous research (e.g., Mrug et al. 2012) has suggested that children with ADHD symptoms tend to have friends who exhibit high levels of behavioral problems. It is possible that children who exhibit symptoms of ADHD early on are rejected by prosocial peers or school and/or treated differentially by their teachers, and as a result later engage in aggressive or defiant behaviors. Building on these findings, it will be important to further examine the mechanisms linking ADHD symptoms to later externalizing problems.

Our findings here are only partially consistent with those reported by Shevlin and colleagues (2017) who reported significant links between ADHD and externalizing problems (in their case ODD and CD) in both directions from age 7.5 to age 14, thus providing support for reciprocal heterotypic continuity for disorders within the externalizing dimension. However, those authors only measured these disorders at two time points almost 7 years apart. In that sense they examined a form of long-term heterotypic continuity, which may paint a different picture to year-to-year heterotypic continuity examined in our study. A variety of developmental processes, such as negative responses from peers that may exacerbate aggressive reactions may unfold during the span of 7 years that may contribute to these links over a longer period of time.

Notably, similar results to ours were reported in an earlier study by Costello et al. (2003) who, having carried out lagged analyses on a sample of 9 to 16 year olds, found that ADHD earlier in life (9–13) predicted ODD later in life, although the reverse was not the case when controlling for homotypic continuity. Together these findings are consistent with the developmental spectrum model of development (Beauchaine and McNulty 2013) that describes the development of externalizing problems via an ontogenic, or transactional, process. In this process individual vulnerabilities and contextual risk factors influence each other over time and manifest in a range of externalizing difficulties. This further highlights the need for year-to-year (or ‘short term’) heterotypic continuity assessments with the goal of understanding the potentially causal links between different behaviors. Our findings also highlight the importance of targeting these problems at different times during a child’s development. Understanding of these links from childhood through adolescence will aid in the development of targeted intervention programmes and guidelines for timely detection and prevention of mental health difficulties.

Furthermore, because past research has pointed to aggressive behavior and non-aggressive externalizing problems as two distinct constructs (e.g., Burt 2012), we were interested in examining both in relation to ADHD symptoms over time. At first glance, our findings do not provide support for this possibility as the pattern of findings was the same for both in relation to ADHD symptoms. ADHD symptoms consistently predicted both of these externalizing behaviors, but neither of them consistently predicted ADHD symptoms. However, this does not mean that the two are not different constructs. It is possible that they behave similarly in relation to symptoms of ADHD due to a shared third factor such as irritability that may be underlying both the links between ADHD symptoms and aggression and ADHD symptoms and non-aggressive behavior problems.

With respect to the longitudinal links between ADHD symptoms and anxiety/depression our findings were not consistent across time or direction of effects. Specifically, ADHD symptoms at ages 7 and 9 predicted higher level of symptoms of anxiety/depression at ages 8 and 10, respectively. However, symptoms of anxiety/depression at ages 9, 12 and 13 predicted lower levels of ADHD symptomatology at ages 10, 13 and 15, respectively. These findings are again only partially consistent with those reported by Shevlin and colleagues (2017) who reported positive links between ADHD and anxiety/depression from age 7.5 to 14. As noted above, the processes linking problem behaviors year-to-year, as in our study, may be different than those linking behaviors from age 7.5 to 14. These processes need to be explored further in order to better understand these developmental links.

Few previous studies have examined the longitudinal links between ADHD symptoms and internalizing problems. The majority of the available studies have focused on these links in young children. Partly consistent with our results, one such recent study (Wichstrøm et al. 2017) based on a community sample of children in Norway assessed biennially from age 4 to 10 found that ADHD predicted anxiety two years later but not vice versa. In our study anxiety/depression were predictors of lower levels of ADHD symptoms at two time points during adolescence. As Wichstrøm et al.’s (2017) study did not cover this developmental period, this may account for the difference in the findings across the two studies. Notably, unlike the findings related to the links from ADHD to externalizing problems, the links between ADHD symptoms and internalizing problems were not consistent over time. In other words, ADHD symptoms did not consistently predict anxiety/depression, and contrary to expectations anxiety/depression served as a protective factor against ADHD symptoms, particularly in adolescence.

This may be due to the time-specific and time-limited nature of these links. As we found that ADHD symptoms predicted anxiety and depression in younger, but not older, children, one may speculate that not fully mastering self-regulation strategies at a younger age, due to a deficit in executive functioning strategies underlying ADHD, may interfere with children’s ability to read social cues that are important to engage and maintain positive interactions and relationships. Such forms of social isolation may in turn result in symptoms of anxiety/depression. By adolescence, young people may develop strategies to cope with these situations and may adapt to their symptoms of ADHD. In fact, in adolescence, symptoms of anxiety/depression seemed to serve as a protective factor against ADHD symptoms; as well as aggression. It is possible that in adolescence with the growing ability to self-regulate, symptoms of anxiety/depression may mask or inhibit, at least some, symptoms of ADHD and ward against aggression (e.g., Bloemsma et al. 2013).

Interestingly, no sex differences were found in the assessed patterns, thus suggesting that girls and boys from age 7 to 15 follow the same pattern when it comes to the four assessed problem behaviors. The lack of differences in the model fit for girls and boys was consistent with other studies that suggest differences in base rates of behaviors between the two sexes but not in their predictors, outcomes, or comorbidities (e.g., Yoshimasu et al. 2012) in non-clinical community and population-based samples. Sex differences are reported in studies examining ADHD sub-types, for instance more girls are rated to be inattentive and more boys impulsive (e.g., Staller and Faraone 2006) alongside a reported gender bias toward boys being diagnosed with ADHD more frequently (e.g., Mowlem et al. 2019). Therefore, further research is required to shed more light on the symptoms of ADHD sub-types and their links to other problem behaviors over time to better understand these links.

The current findings should be interpreted in the context of some limitations, some of which point to future directions of research. For instance, we only examined a set number of problem behaviors and their developmental links with ADHD symptoms. Moreover, our measure of ADHD symptoms relied on eight items, thus it did not assess the full range of 18 symptoms of ADHD. Arguably, inclusion of a broader range of ADHD symptoms as well as symptoms of additional disorders and examination of their predictive links over time would further illuminate the longitudinal dynamics of ADHD symptoms with other disorders. In addition to the currently examined disorders, future studies could, for instance, include other neurodevelopmental disorders, such as learning disorders and autism spectrum disorder, in order to tease out the potential causes and developmental links between these disorders (Dewey 2018). In this study symptoms of anxiety and depression were combined thus precluding us from examining possible differences in their developmental links and their links to other symptoms over time. Future studies should examine the links between other disorders and symptoms of depression and anxiety separately to allow for identification of possible developmental differences.

Furthermore, because we were interested in examining a wide range of ADHD symptoms, including those that are sub-clinical, we relied on a general measure of psychopathology. The degree of construct validity of this measure with established measures of ADHD is unknown. Therefore, the results of this study should be interpreted with this in mind. Future studies may utilize ADHD measures and examine the presence of symptoms across different contexts. Similarly, future research may examine the longitudinal links between Inattention and Hyperactivity, alongside other symptoms, as it is possible that the patterns of reciprocal influences may be unique to each. For example, one may predict a potentially stronger relation between anxiety/depression and ADHD inattention than ADHD hyperactivity. This study arguably also lacks a multi-informant perspective approach, as data were gathered from teachers only. However, agreement is typically modest between different informants’ reports for adult, as well as for child psychopathology. It is often reported that discrepancies between informants may reflect variations in functioning in different contexts (Murray et al. 2018). Some research suggests that teacher reports of externalizing behavior are more reliable than those of children or parents. This is because children and young people spend significant amounts of their time at school thus giving teachers an opportunity to observe their behavior. However, teacher reports have been shown to be less sensitive in identifying internalizing problems, thus it is possible that our study underestimated the levels of these difficulties in our sample.

Despite its limitations, our study also has several strengths. Previous studies have predominantly focused on the age range from infancy to early childhood, and/or only included assessments at two points (ages) in development with varied time-periods between them. However, given the implication of ADHD symptoms in school attainment, socialisation processes and peer relationships in adolescence, we expanded previous research by assessing the problem behaviors at ages 7, 8, 9, 10, 11, 13, and 15. This allowed for a fine-tuned insight into the links between problem behaviors over time but also, importantly, from year-to-year. In addition, because our study relies on continuous measures capturing symptoms of psychopathology and draws on a community-based cohort sample, it allowed us to report on a wide range of severity in problem behaviors. Furthermore, the study was carried out utilizing a normative sample of young people thus represents a broader range of problems and provides a better view of these patterns in the general population. As such, the results may be more reliably used to inform primary prevention practices targeting young people in the general community.

In conclusion, our study provided support for consistent concurrent comorbidity and homotypic continuity from age 7 to 15. However, we only found limited support for heterotypic continuity when assessed year-by-year from entry to school to adolescence. Importantly, ADHD symptoms at each assessment period emerged as risk factors for both types of externalizing behaviors in subsequent years, but not for anxiety/ depression. Our findings highlight the importance of focusing on ADHD symptoms to inform prevention and early intervention practices specifically for externalizing behavior problems for both girls and boys. The findings also pointed to the importance of assessing these behaviors/ symptoms regularly at different developmental stages. Future studies should focus on identifying the mechanisms through which the links between ADHD symptoms and other problem behaviors are formed as well as on exploring the year-to-year predictors of both externalizing and internalizing problems.
